# The Neuroimmune Role of Intestinal Microbiota in the Pathogenesis of Cardiovascular Disease

**DOI:** 10.3390/jcm10091995

**Published:** 2021-05-06

**Authors:** Andrey V. Suslov, Elizaveta Chairkina, Maria D. Shepetovskaya, Irina S. Suslova, Victoria A. Khotina, Tatiana V. Kirichenko, Anton Y. Postnov

**Affiliations:** 1 I.M. Sechenov First Moscow State Medical University, Ministry of Health of Russia, 8-2 Trubetskaya Str., 119992 Moscow, Russia; suslov_a_v@staff.sechenov.ru (A.V.S.); chairkin@rambler.ru (E.C.); mr.mary170600@gmail.com (M.D.S.); 2 Central State Medical Academy of the Administrative Department of the President of the Russian Federation, 19-1A Marshal Timoshenko Str., 121359 Moscow, Russia; suslova.is@rambler.ru; 3 Research Institute of Human Morphology, 3 Tsyurupy Str., 117418 Moscow, Russia; nafany905@gmail.com (V.A.K.); anton-5@mail.ru (A.Y.P.); 4 Institute of General Pathology and Pathophysiology, 8 Baltiyskaya Str., 125315 Moscow, Russia; 5 National Medical Research Center of Cardiology, 15A 3-rd Cherepkovskaya Str., 121552 Moscow, Russia

**Keywords:** intestinal microbiota, cardiovascular disease, epithelial intestinal barrier, hypothalamic-pituitary system

## Abstract

Currently, a bidirectional relationship between the gut microbiota and the nervous system, which is considered as microbiota-gut-brain axis, is being actively studied. This axis is believed to be a key mechanism in the formation of somatovisceral functions in the human body. The gut microbiota determines the level of activation of the hypothalamic–pituitary system. In particular, the intestinal microbiota is an important source of neuroimmune mediators in the pathogenesis of cardiovascular disease. This review reflects the current state of publications in PubMed and Scopus databases until December 2020 on the mechanisms of formation and participation of neuroimmune mediators associated with gut microbiota in the development of cardiovascular disease.

## 1. Introduction

Cardiovascular disease (CVD) is still the leading cause of death and disability in developed countries all over the world. Approximately one in three deaths in the United States and one in four deaths in European countries are due to CVD [1]. Moreover, the widespread cardiovascular risk factors such as metabolic syndrome, diabetes mellitus, obesity, and sex steroid hormones metabolism disorders define the necessity of searching for strategies that are more effective in the prevention of cardiometabolic disorders [2,3,4]. Recently, remarkable interest has been focused on the role of the human gut microbiota in the pathogenesis of CVD, and modern knowledge allows developing new personalized approaches to the prevention and treatment of CVD.

Atherosclerosis is one of the key factors in the development of CVD. Bacterial DNA presented in the intestinal microbiota was isolated from atherosclerotic plaques [5]. Moreover, another research demonstrates that *Akkermansia muciniphila* rebuilds the barrier function of the intestines and provides antiatherogenic effect [6]. Changes in the composition of gut microbiota and its metabolic status have also been associated with the risk of CVD development. In particular, trimethylamine N-oxide (TMAO) is considered to be a potential risk factor for atherosclerosis and cardiometabolic diseases [7,8,9]. Some research showed that gastrointestinal microbiota is connected with blood pressure regulation [10,11]. The increase of butyrate-producing bacteria from the genus *Odorbacter* count is associated with decrease of blood pressure among women with obesity [12,13]. More scientific evidence confirms the role of intestinal microbiota in the pathogenesis of heart failure. It was found that patients with heart failure and peripheral edema had higher concentrations of endotoxins and inflammatory cytokines in blood plasma than patients without edema [14,15].

Animal researches support the connection between obesity and the increase of gastrointestinal bacteria from phyla *Firmicutes* and *Bacteroidetes.* The decrease in short-chain fatty acids (SCFA) producing bacteria indicates a disorder of glucose homeostasis [16,17]. Animal studies also confirm that gastrointestinal bacteria regulate blood lipids level in human organisms. Bile acids synthesized by gastrointestinal bacteria, move from the intestine into bloodstream and modulate the hepatic and system metabolism of lipids and glucose [18,19]. 

Nowadays, the bidirectional relationship between the gastrointestinal microbiota and the nervous system, which is considered as microbiota–gut–brain axis, is being actively studied [20,21,22,23]. In this review, we have reflected the current state of publications in the Pubmed and Scopus databases until December 2020 on the formation mechanisms of neuroimmune mediators of intestinal microbiome origin and their role in the pathogenesis of CVD.

## 2. Human Intestinal Microbiome Variability

The gut microbiome develops and matures during growing up. The child’s first contact with microbes supposedly should happen after the rupture of the sterile amniotic sac. However, it was determined that the placenta and the infant’s first stool contained a full set of microbes [24,25,26] and it was shown that the traced strain of *Enterococcus faecium* could enter through the umbilical cord in mice [27]. The way of parturition is one of the key factors which defines the child’s gastrointestinal microbiota composition [28,29,30,31]. However, some authors conclude that during the first 6–12 weeks of life, an infant’s microbiota undergoes a considerable reorganization, which is mainly defined by the child’s living conditions but not by the way of parturition [32,33]. 

The intestine microbiota of an adult consists of the common bacterial core which includes two basic phyla—*Firmicutes* and *Bacteriodetes*, while the other part of the gastrointestinal microbiota is quite diverse. This variety often includes less common samples such as *Proteobacteria*, *Verrumicrobia*, *Actinobacteria*, *Fusobacteria*, *Cyanobacteria*, and *Archaea* [34]. The group number changes through the gastrointestinal tract under the influence of different factors and accessibility of nutrients [35].

Therefore, the difficulty in predicting the pathogenic role of the particular gastrointestinal bacteria is due to many factors, namely, different phases of gastrointestinal microbiota development, widely varied medicament concentrations in the organism, including antibiotics, the level of medicine development, living conditions, co-morbidities, and many others [36,37,38,39,40]. Consequently, any attempts to differentiate any bacteria that are responsible for the development of the disease are associated with a large number of objective difficulties.

## 3. The Anatomy–Functional Connection between the Autonomic Nervous System and CVD

The functioning of the cardiovascular system (CVS) is under constant and dynamic control of the autonomic nervous system (ANS). ANS adapts CVS to the effects of external and internal environmental factors. The ANS control over the CVS is carried out using cardiovascular neuroaxis which is presented at multiple levels of integrative centers. In particular, the intrinsic cardiac nervous system (ICNS), represented by ganglionated plexi, functions at the level of heart [41]. ICNS is connected with the sympathetic paravertebral ganglia, the extrathoracic cardiac ganglia, and central nervous system as well as with parasympathetic system and provides coordinated response of the heart to different stimuli [42]. Moreover, the mechanism of CVS afferent control by the ANS includes baroreceptors, chemoreceptors, and mechanoreceptors, which are located in the wall of large vessels, primarily in the aorto–carotid zone [43]. The parasympathetic nervous system interacts with ICNS via preganglionic parasympathetic fibers from the cervical vagus nerve, providing a coordinated response in the heart [42,44]. That is, the response of the CVS to external and internal factors is determined by the work and functional status of the ANS. The balance of afferent and efferent reactions of the ANS is carried out to ensure the necessary functional status of the CVS under conditions of rest, physical activity or stress.

Stress is the universal and obligatory component of CVD pathogenesis. Stress accompanies the CVD during the whole period of disease independently from the particular CVD nosology. In different periods, stress can act as an acute attacking factor, which leads to irreversible changes in the organism, organs, and tissues, or it can be a factor, which compensates the organism’s dysfunction during CVD [45,46]. Stress-induced cardiovascular reactions are the result of dynamic regulation of the efferent and afferent pathways of the ANS and, at the same time, the work of the hypothalamus–pituitary–adrenal axis. Cardiovascular changes due to autonomic and neuroendocrine regulation are necessary for the organism to respond to expected or current needs [47]. At the same time, in the course of chronic stress, cardiovascular reactions initiate pathophysiological changes in the CVS, such as the progression of atherosclerosis, the development of hypertension, myocardial infarction, heart remodeling, and others [48,49]. 

Thus, stress development should be considered as a mechanism which performs the filigree adjustment of an organism’s homeostasis towards the development of irreversible changes or the formation of compensatory and adaptive mechanisms during CVD. Consequently, stress control is a logical and reasonable task in CVD patient treatment.

## 4. Pathophysiological Mechanisms of Intestinal Barrier Insufficiency during CVD-Associated Stress

**Figure 1:**
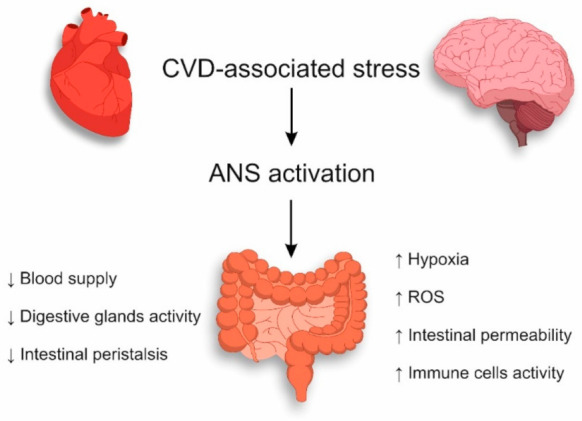
Mechanisms of intestinal epithelium damage during CVD-associated stress.

The stress associated with CVD affects the whole organism, including the gastrointestinal tract, by activating the sympathetic division of the ANS. Under the influence of ANS, the decreased blood supply to the intestines with the inhabited microbiota reduces the activity of the digestive glands, and intestinal peristalsis in the gastrointestinal tract slows down [50]. The above-mentioned mechanisms determine further disturbance of the intestinal epithelium due to CVD-associated stress (Figure 1). 

The intestinal wall is innervated by adrenergic sympathetic nerve fibers which increase water and natrium absorption during the stimulation [51,52] that accompanies an increase of the intestinal permeability. At the same time, the mucus production by intestinal epithelial goblet cells decreases under the influence of the vagus nerve in the large intestine [53]. It is noteworthy that, on the one hand, mucus forms a protective shield for the intestinal epithelium from commensals and their metabolites and, on the other hand, the mucus blocks an untimely activation of immune cells. Consequently, the reduction of the mucus layer and the increase of the intestinal wall permeability can lead to the disorder of intestinal bacteria and spatial segregation of the intestinal epithelial cells [54,55]. It was shown in Wistar rats that limited nesting stress at early post-natal period leads to hypercorticosteronemia, increased intestinal permeability, and decrease of fecal microbial diversity with disbalance of gut microbiota composition [56]. The decrease of intestinal blood supply is not only due to the effect of the sympathetic division of the ANS but also due to the pathogenetic influence of CVD. It was shown in several studies that blood-supply failure in the intestine accompanies many types of CVD: myocardial infarction, severe atherosclerosis, chronic heart failure, diabetes mellitus, and obesity [57,58,59]. Thus, blood-supply failure in the intestine during CVD is determined simultaneously by several mechanisms. The decrease of intestinal blood supply is accompanied by tissue hypoxia. The intestinal mucus membrane is mostly sensitive to hypoxia [60]. It is an anatomical structure which supports the mucus layer as well as spatial segregation of microbiota from subepithelial tissue. During hypoxia, glucose transformation in aerobic and anaerobic catabolism cycles impairs biological synthesis of energy at intermediate stages. This results in the release of reactive oxygen species (ROS) [61,62]. Reperfusion increases damaging effects of ischemic injury due to accumulation of activated immune cells and generation of ROS [63]. ROS have a high reactivity towards proteins, lipids, carbohydrates, and nucleic acids, that leads to intestinal epithelium integrity damage [64,65]. As for the relationship between gut microbiota and ischemic intestinal injury, it was shown in a rat model that intestinal ischemia–reperfusion injury caused significant changes in the gut microbiome with an increase of the number of Escherichia coli and Prevotella oralis, followed by the enhancing of Lactobacilli in the healing stage [66]. At the same time, it was demonstrated in a model of rats with acute myocardial infarction that gut microbiota alterations cause the development of intestinal inflammation and apoptosis, that is, not only intestinal ischemia leads to imbalance of gut microbiome but vice versa—changes in the microbiome lead to gut damage [67].

In addition to the mucus layer, the intestinal epithelial layer plays an important role in providing functions of the intestinal barrier. Intestinal epithelial layer consists of epithelial cells connected with tight junction proteins, in particular, claudins, occludins, cadherins, and adhesion molecules [68,69]. Tight junction proteins are important as an element in the intestinal barrier in the structure of the gut–brain axis. It was shown that ghrelin, a brain–gut peptide, attenuates intestinal barrier dysfunction activating tight junction proteins zonula occludens-1 and claudin-5 after intracerebral hemorrhage in animal model [70]. Some studies demonstrate the relationship of gut microbiome changes with intestinal barrier injury through alteration of tight junction proteins. For example, Lactobacillus plantarum enhances the epithelial barrier stimulating expression of genes involved in the signaling pathway of tight junctions zonula occludens-1, zonula occludens-2, and occludin [71]. The same effects were demonstrated in the other study in a mice model, where treatment with a mixture of Lactobacillus, Bifidobacterium, and Streptococcus increased the expression of tight junctions zonula occludens-1 and claudins [72]. Alterations of tight junction integrity can lead to increased influx of bacteria or bacterial metabolites associated with an impaired metabolic host status, manifested in cardiometabolic disease [73].

## 5. Intestinal Microbiota in Neuroimmune Network Formation

The intestinal epithelium and mucus barrier is located between the intestinal environment, intestinal bacteria, and the immune system. It is known that the intestinal epithelial layer includes different types of cells: enterocytes, goblet cells, enteroendocrine cells, Paneth cells, tuft cells, and M-cells [74]. Multiple professional immune cells, such as lymphocytes, dendritic cells, and macrophages, are located in immediate proximity to the surface of the intestinal mucus membrane. Intraepithelial lymphocytes (IELs), the first immune cells responding to pathogenic factors, invade the epithelium and spread dendrites to detect luminal antigens [75]. Other cells are located in organized lymphoid structures, such as Peyer’s patches and cryptopatches, or are dispersed inside the lamina propria [76,77,78,79].

Similarly to professional immune cells, such as macrophages and dendritic cells, intestinal epithelial cells express innate immune receptors like pattern recognition receptors, including Toll-like receptors (TLR) and nucleotide-binding proteins, containing oligomerization domain (NOD). Synthesis of antimicrobial molecules by Paneth cells is regulated by TLR4/MyD88 and NOD2 signal transmissions, which are controlled by intestinal microorganisms [80,81,82]. TLR plays a fundamental role in the innate immune system by activating pro-inflammatory signaling pathways in response to microbial antigens.

Intestinal immune cells support the barrier function of the intestinal mucus membrane through cytokines or with direct cell junctions. So, IL-17 and IL-22 produced by Th17 cells or Type 3 innate lymphoid cells (ILC3), increase AMP and Reg3 family protein secretion by intestinal epithelial cells [83]. Moreover, IL-6 produced by intraepithelial lymphocytes, enhances intestinal epithelial cell proliferation and promotes the repair of the mucus membrane after injury [84]. However, other pro-inflammatory cytokines, such as TNF-α and IFN-γ, inhibit epithelial cell proliferation by the suppression of β-catenin/T cell factor (TCF) signal transmission [85,86].

Intestinal epithelial cells also modulate the host immune response by the secretion of cytokines and chemokines. During the stimulation of the intestinal endothelium with flagellin proteins of the gram-negative bacteria Escherichia and Proteus, TLR5 / MyD88 signaling promotes the production of IL-8, which recruits neutrophils into the lamina propria [87,88]. Cholecystokinin, glucagon-like peptide (GLP), and serotonin are secreted by intestinal endocrine cells and influence the activity of the immune system in the gut [89]. Cholecystokinin regulates the differentiation and production of cytokines by CD4+-cells and B-cells [90,91]. It is notable that the decrease of the activity of digestive glands regulated by the sympathetic nervous system indirectly influences the activity of the immune cells.

It is interesting that the microbiota’s influence on the epithelial intestinal barrier is determined not only by the immune component but also by other effects. In particular, short-chain fatty acids (SCFA) synthesized by gut microbiota are used as a source of energy for the epithelium and strengthen the epithelial barrier indirectly [92,93]. The microbial metabolite indole has a defensive barrier effect through the activation of the pregnane-X receptor and increases the secretion of glucagon-like peptide-1 [94].

The disability to save complex anatomical and functional characteristics of the intestinal epithelium decreases the antimicrobial, immunoregulatory, and regenerative capability of the epithelial barrier. The destruction of the mucous membrane leads to the translocation of the commensal bacteria and their metabolites from the intestine lumina into the subepithelial tissue, leading to the secretion of pro-inflammatory cytokines [95,96]. In turn, this causes the dysfunction of the organ and is accompanied by the inflammation of the intestinal mucosa [97,98].

Today, there are more and more proofs that metabolites of the intestinal bacteria reach the circulation through the destroyed intestinal barrier during inflammation [98,99,100,101]. Thaiss and co-authors highlight three factors affecting microbiome-mediated diseases at the same time [102]. Firstly, metabolites of the intestinal bacteria are permanent activators of the chronic immune reactions, which cause persistent inflammation in the intestine as well as in the whole organism. Secondly, dysbiotic disturbance of intestinal microecology during the maturation period of the innate immune system leads to impaired immunological tolerance, which subsequently manifests in autoimmune and auto-inflammatory disorders. Thirdly, the microbiome can influence immunological factors that control tissue-specific immunity in the distance from intestine. Therefore, the authors conclude that the intestinal microbiome provides pathophysiological mechanisms in normal and pathology. The barrier function of the intestinal epithelium is impaired during CVD, along with a disturbance of the spatial separation of the gut microbiota, synthesis of inflammatory mediators by immune cells, and their further penetration into all tissues of the organism.

Considering the role of the gut microbiota in the formation of the neuroimmune network, it is worth mentioning that the mechanisms underlying the formation of the microbiota–gut–brain axis are being actively studied. Brain-derived neurotrophic factor (BDNF) is a stress protein, a member of the neurotrophin family, which increases the resistance of neurons in the brain to dysfunction and provides the plasticity of the nervous system. A wide spectrum of processes are controlled by BDNF, including the involvement of microbiota–gut–brain axis in the pathogenesis of cardiometabolic disease [103]. It was demonstrated that BDNF signaling may mediate effects of intermittent fasting on glucose regulation and cardiovascular function [104]. Moreover, it was shown that treatment with high doses of probiotics can modulate behavior in Zebrafish, causing significant changes in the expression of some brain-relevant genes, such as BDNF [105]. Thus, BDNF may represent a molecular mechanism underlying the microbiota–gut–brain axis.

## 6. The Neuroimmune Axis: Microbiota–Intestine–Brain-CVD

Hypoxic injury of the intestinal mucus membrane, microbiota’s displacement to the subepithelial tissue, destruction of the intestinal epithelium’s barrier function, intestinal bacteria metabolites, and inflammation cytokines’ synthesis make the intestine the greatest endotoxin source. Inflammatory mediators reach centers of the nervous system through systemic blood and lymph circulation [67,106,107].

The blood–brain barrier (BBB) forms during gestation and serves as a selective filter between the brain and the blood circulatory system. The importance of the intestinal microbiota and microbial metabolites in the BBB formation was confirmed on gnotobiotic mice. In the absence of the intestinal microorganisms, the mice’s BBB becomes permeable compared with the BBB of normal animals [108].

It was discovered that the lymphatic system of the brain drains to the cerebrospinal fluid, goes to the subarachnoid space, and further travels to deep cervical lymph nodes. The lipids’ solubility, the proteins’ tertiary structure, the concentration, the molecular mass, and the charge of compounds determine the passage of mediators from the peripheral blood supply and the lymphatic system to the brain [109]. Cytokines presented in the peripheral blood are mainly hydrophilic and can modulate immunological functions in the nervous system [109,110]. It was also shown that the intravenous injection of indol, which is similar to the product of bacterial metabolism of tryptophan, allows overcoming the BBB [111]. Neuro-inflammatory effects of LPS act through TLR activation in peripheral tissues, causing secondary effects in the nervous system by BBB-positive pro-inflammatory cytokines [112,113,114].

The BBB and the lymphatic vascular system are considered as the entry for the signals going to the brain. For example, circulating immune cells and inflammation mediators (including hormones and neurotransmitters of both the host and bacteria) along with the vagus nerve stimulation represent mechanisms which contribute to direct or indirect microbial signals’ transmission from the intestine to the brain [115,116,117].

Inflammatory cytokines are also an important factor activating the central nervous system as the response to various stimuli, including pro-inflammatory cytokines which activate the hypothalamic–pituitary–adrenal axis during intestinal pathology. Cortisol and the proinflammatory cytokines interleukin (IL)-6 and IL-8 was significantly increased in patients with irritable bowel syndrome [118]. IL-1α cytokine stimulates the whole glucose metabolism in the organism on the central nervous system level [119]; IL-6, IL-1, TNF-α, and IFN cytokines stimulate the hypothalamic–pituitary–adrenal axis (HPA) independently of each other [120,121,122]. Besides inflammatory cytokines, prostaglandins synthesized in the cyclooxygenase system during the inflammation take part in the HPA axis activation [122]. Multiple researches studied the role of inflammatory cytokines (TNF-α, IL-1, and IL-6) in the HPA axis activation. The injection of any inflammatory cytokines stimulates the HPA axis and leads to an increase of the circulating corticosterone level [120,123]. It is noteworthy that the blockade of any cytokines does not block the HPA axis activation after the penetration of LPS, that is, if the intestinal epithelium barrier function brakes and LPS enters the bloodstream, then the duplicating effect of activation of HPA axis by cytokines is implemented [124,125]. Consequently, all inflammatory mediators promote HPA axis activation, while the blockade of any single cytokine cannot decrease the HPA axis stimulation because of the duplicating effects of each other [122,126].

Thus, the activation of the hypothalamic–pituitary–adrenal axis is one of the fundamental brain-mediated responses to disease. The HPA axis is considered as a basis of the neuroendocrine system that regulates the homeostasis in the organism under the influence of psychological and physical stress, including infections, promoting adequate reaction to the stress [127,128,129].

The considered mechanisms are of great importance in chronic stress. Since the threshold of emotional arousal is insufficient for the formation of stress during CVD, a full value stress response forms in the nervous system with the subsequent activation of the sympathetic division of the ANS through persistent activation of the HPA with inflammatory mediators from the intestine [130]. It is noteworthy that the whole complex of pathological changes in the organism develops by the acute stress pathway, while the emotional component (emotional stimulus) corresponds with the chronic stress threshold or is absent completely. This question requires further study. Therefore, reviewed mechanisms have an activating influence on nervous system centers, including ANS centers, which in turn innervate internal organs, including the intestine inhabited by microbiota.

It was shown that bacterial components of the gut microbiota in patients with attention deficit/hyperactivity disorder (ADHD) are associated with changes in the brain’s structure and functions as well as with behavior reactions [131,132]. The study results were based on intestinal microbiota transplantation.

Taking into account modern knowledge about intestinal microbiota and its connection with the disorder of the nervous system development through the gut–brain axis [133,134,135], it can be concluded that during CVD, mediators from the intestine enter the brain with the bloodstream and lymph and activate hypothalamus nuclei. Then, as far as the hypothalamus is a suprasegmental integral center of the ANS, the sympathetic division of the ANS is activated [136,137]. Thus, mediators from the intestine reach suprasegmentals centers of the ANS and activate the work of sympathetic and parasympathetic divisions, thereby closing the pathological circle of the intestinal microbiota participation in the CVD pathogenesis. Numerous publications demonstrate that an increase in microbiota-mediated inflammatory mediators aggravates the course and prognosis of disease in CVD [138,139,140,141]. It is also found that the correction of the intestinal microbiota in patients with CVD improves the prognosis of the disease. Prescribing in the complex therapy preparations that increase the number of bacteria from genus *Akkermansia*, *Bifidobacteria*, *Lactobacillus*, *Bacteroides*, and *Prevotella* ameliorates the course of CVD [142,143,144]. It is known that bacteria from *Bifidobacteria* and *Lactobacillus* genus provide a local anti-inflammatory effect on the intestinal wall. The recovery of the barrier function of intestinal epithelium occurs because of the decrease of inflammation in the intestinal wall, meaning that the level of inflammatory mediators decreases in systemic circulation and consequently their activating effect on the nervous system reduces [145,146,147]. In particular, hypertension is associated with disturbance of gut microbiome and dysregulation of gut–brain axis. It was demonstrated in a model of hypertensive rats that long-term kefir treatment reduced IL-6 and TNF-α protein density and abolished the microglial activation observed in the hypothalamic paraventricular nucleus and rostral ventrolateral medulla defending cardioregulatory nuclei from gut-mediated inflammation that provides the hypotensive effect of kefir [148]. Some studies in mice model of ischemic stroke or cerebral ischemia demonstrate that ischemic stroke brain injury promotes the development of gut dysbiosis with increase pro-inflammatory responses and infiltration of brain structures with cytokines, chemokines and immune cells that is associated with poor prognosis [149].

**Figure 2:**
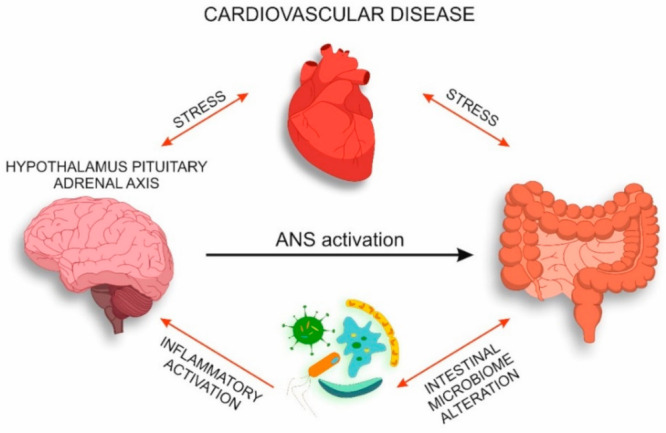
Interaction of intestinal microbiota and nervous system in CVD.

The mechanisms considered in the present review form a pathologic vicious circle where intestinal microbiota is involved in the pathogenesis of CVD and determines the inflammatory activation of HPA axis (Figure 2).

Some studies investigated that microbiome-targeted preparations ameliorate the course of CVD, decrease the progression of atherosclerosis and risk of major CVD complications [150,151]. In the context of this review, we can suppose that beneficial cardioprotective mechanisms of microbiome-based treatment are due to its influence on the microbiome–gut–brain axis. It was shown in rats with induced myocardial infarction by occluding the left anterior coronary artery, administration of probiotics based on combination of Lactobacillus helveticus and Bifidobacterium longum reduces Bax/Bcl-2 (pro-apoptotic/anti-apoptotic) ratio and caspase-3 (pro-apoptotic) activity in the amygdala and dentate gyrus in comparison with the placebo group decreasing the predisposition of apoptosis in different cerebral regions associated with myocardial infarction [152]. Another study in mice demonstrates that antibiotic administration modulating gut microbiota reduces LPS levels and neuroinflammation in the ischemic brain after experimental stroke [153]. A study in patients with coronary artery disease found that probiotic Lactobacillus Rhamnosus in complex with prebiotic inulin provides beneficial effects on depression, anxiety, and inflammatory biomarkers [154].

## 7. Conclusions

The literature review elucidates the potential neuroimmune role of the intestinal microbiome in the pathogenesis of CVD. At the initial stage of CVD the role of the intestinal microbiota in its pathogenesis is of secondary significance, meaning that the qualitative and quantitative changes in bacteria are not as important as at the following stages. However, later, when the intestinal microbiota determines the level of inflammatory activation of the hypothalamus–pituitary–adrenal axis, changes in the intestinal microbiota become significant for CVD development. During CVD progression intestinal bacteria are in close interaction with the pathologic processes developing in the intestinal wall and become one of the key elements in CVD pathogenesis. In this regard, attempts to determine the intestinal bacteria most involved in the process of CVD progression can be an important step for the development of new relevant methods for the diagnosis, prevention, and therapy of CVD.
